# A definitive haplotype map of structural variations determined by microarray analysis of duplicated haploid genomes

**DOI:** 10.1016/j.gdata.2014.04.006

**Published:** 2014-04-24

**Authors:** Tomoko Tahira, Koji Yahara, Yoji Kukita, Koichiro Higasa, Kiyoko Kato, Norio Wake, Kenshi Hayashi

**Affiliations:** a Medical Institute of Bioregulation, Kyushu University, Fukuoka, Japan; b Biostatistics Center, Kurume University, Kurume, Japan; c Research Institute, Osaka Medical Center for Cancer and Cardiovascular Diseases, Osaka, Japan; d Center for Genomic Medicine, Kyoto University, Kyoto, Japan; e Graduate School of Medical Sciences, Kyushu University, Fukuoka, Japan

**Keywords:** Complete hydatidiform moles, Definitive haplotypes, Single nucleotide polymorphism, Copy Number Variation, LD-bin

## Abstract

Complete hydatidiform moles (CHMs) are tissues carrying duplicated haploid genomes derived from single sperms, and detecting copy number variations (CNVs) in CHMs is assumed to be sensitive and straightforward methods. We genotyped 108 CHM genomes using *Affymetrix SNP 6.0* (GEO#: GSE18642) and *Illumina 1 M-duo* (GEO#: GSE54948). After quality control, we obtained 84 definitive haplotype consisting of 1.7 million SNPs and 2339 CNV regions. The results are presented in the database of our web site (http://orca.gen.kyushu-u.ac.jp/cgi-bin/gbrowse/humanBuild37D4_1/).

**Table:** 

Specifications	
Organism/cell line/tissue	Homo sapiens/complete hydatidiform moles (CHMs)
Sex	Duplicated haploids whose genomes are from single sperms harboring X
Sequencer or array type	*Affymetrix SNP 6.0* and *Illumina 1 M-duo*
Data format	*Affymetrix* Raw data: CEL files, normalized data: SOFT, MINIML and TXT *Illumina* Raw data: GSE54948_ signal_intensities.txt.gz, normalized data: SOFT, MINIML, TXT and GSE54948_matrix_processed.txt.gz
Experimental factors	Single nucleotide polymorphism (SNP), copy number variation (CNV), LD-bin, CNV segments, CNV regions, definitive haplotypes
Experimental features	Whole genome SNP/CNV haplotyping of 84 duplicated haploid samples
Consent	All patients (donors) gave their written informed consent before study entry.
Sample source location	Japan

## Direct link to deposited data

http://www.ncbi.nlm.nih.gov/geo/query/acc.cgi?acc=GSE18642

http://www.ncbi.nlm.nih.gov/geo/query/acc.cgi?acc=GSE54948

## Experimental design, materials and methods

### Samples

Complete hydatidiform mole tissues dissected from patients and the blood sample of one patient served as sources of DNAs for array hybridization experiments as described previously [1]. The informed consent was obtained from each donor. This study was approved by the Institutional Review Board (Ethical Committee of Kyushu University).

### SNP genotyping

The raw data files of *Affymetrix SNP 6.0* arrays (CEL files) and sample attribute files of 94 CHM samples and one blood sample that has passed quality control in the previous study [1] were reanalyzed by *Birdseed v2* of *Geotyping Console 4. 1. 1. 834* (*GTC 4.1*), together with CEL files and sample attribute files of 45 *HapMap-JPT* samples (obtained from *Affymetrix*). The locations of markers in genome coordinate of *GRCh37* were according to *GenomeWideSNP_6.na32* that was obtained from *Affymetrix*. A total of 905,025 SNP genotypes (excluding chromosome Y and mitochondria) were obtained, at an initial average call rate for the 94 CHMs of 99.2%.

Array hybridization experiments using *Illumina 1 M-duo* was performed for 98 CHM samples that included the 94 samples and one blood samples mentioned above by previously described procedures [1]. The genotypes were called using *GenTrain 2.0* cluster algorithm of *Genome Studio 2011.1*, *Illumina. Human1M-Duov3_H.egt* (based on *GRCh37*) was used as the manifest file and *Human1M-Duov3_H.bpm* as the cluster file. The initial average call rate was 99.5%.

### Copy number analysis

The CEL files of *Affymetrix* arrays were subjected to *Copy Number/LOH analysis* module of *GTC 4.1* without regional GC correction. The 94 CHM samples, one blood sample mentioned above and four male samples from *HapMap JPT* (*NA18940*, *NA18943*, *NA18944* and *NA18945*) served as references to obtain “Log2Ratio” (abbreviated as log2R in this paper) data. Then, the data of markers on chromosome Y and mitochondria were excluded and the remaining data were exported as *CNCHP.txt*. The “log R Ratio” (abbreviated as logRR in this paper) data of *Illumina* arrays were calculated by *Genome Studio 2011.1* using the cluster file (*Human1M-Duov3_H.bpm*) as a reference.

## Results and discussion

### SNP genotyping of haploid samples

**Fig. 1:**
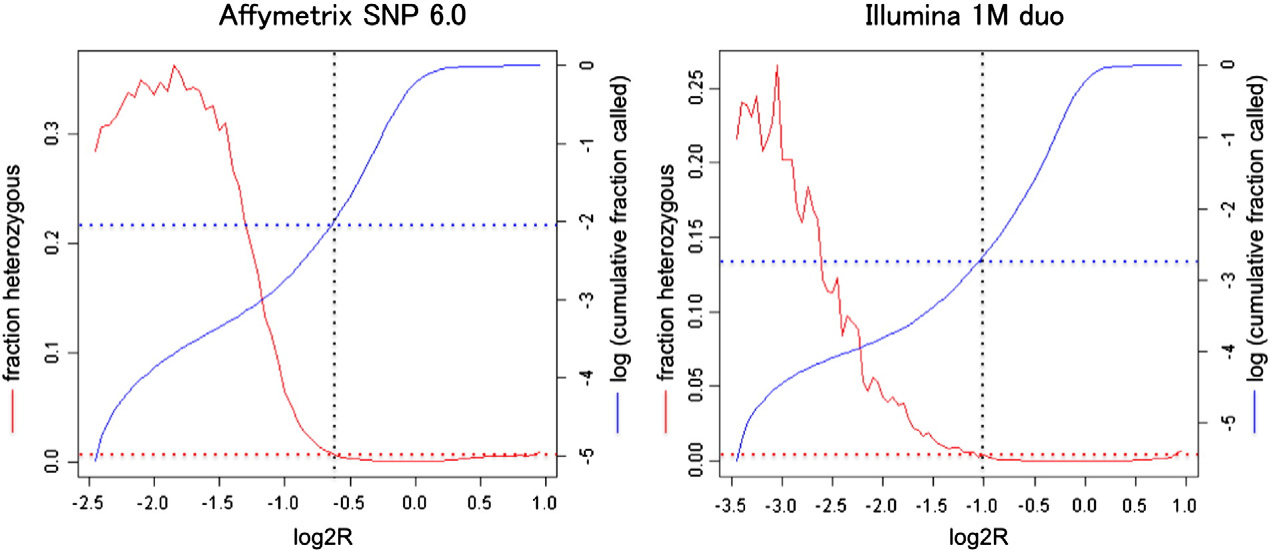
Increased heterozygosity of calls at a low signal intensity. The genotype calls at the relative signal intensity where heterozygosity was approximately 1% (horizontal red dotted lines) or greater were regarded to contain significant fraction of unreliable calls. Blue horizontal lines indicate the fraction of cumulative calls at the reliability thresholds.

CHM genomes are supposed to be genome-widely homozygous. However, the genotypes obtained by the two systems revealed small fractions (0.27% of *Affymetrix* call and 0.01% of *Illumina* call) of heterozygous calls. The dramatic increase of heterozygous calls for the markers at lower relative signal intensities (log2R of *Affymetrix* arrays and logRR of *Illumina* arrays) indicated that the calls were falsely made for the markers at (homozygously) deleted regions where no genotypes should be called, although some of them might be ascribed to the markers in divergent paralogous regions (Fig. 1). These findings provided us an additional quality control measure of SNP genotype calling, that was, forcing all calls at log2R < − 0.6 (0.88% of *Affymetrix* calls), or logRR < − 1 (0.17% of *Illumina* calls) to no-calls. We also removed 164 SNPs in *Illumina* calls, because they were duplicated (i.e., two SNP at the same position). Subsequently, SNPs with call rate less than 90% were removed. After these quality control steps, 84 CHMs, whose SNP genotypes were called at greater than 96% by both platforms, remained.

**Fig. 2:**
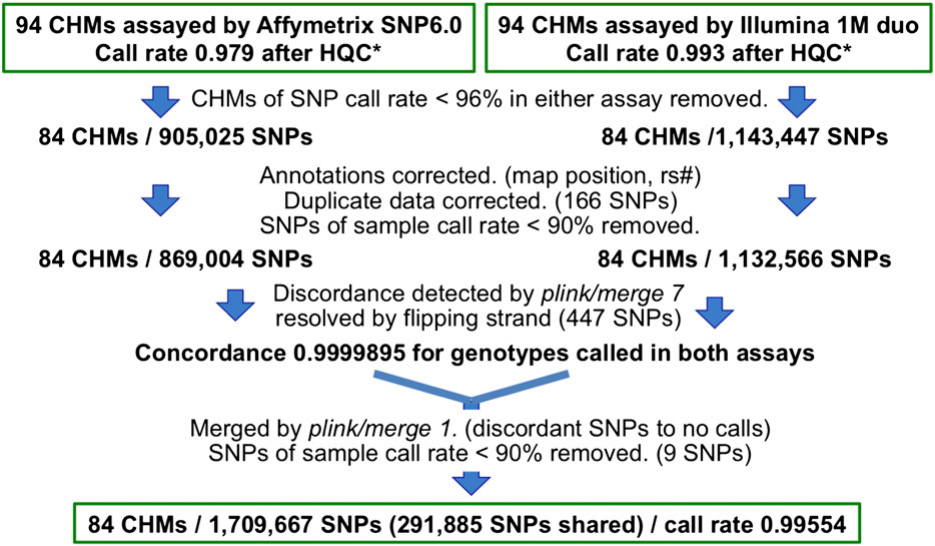
Overview of SNP genotyping and its quality control. *HQC: haploid quality control, that is, heterozygous calls and weak signal calls were forced to no calls. See text for detail.

The genotypes of both platforms were compared using merge function of *PLINK* program version 1.07 [2], that revealed considerable strand inconsistencies between the two platforms. We flipped the strands of *Illumina* data for these SNPs to resolve inconsistency with *Affymetrix* annotation. After these corrections, the fraction of discordant calls was 1.05 × 10^− 5^, which were forced to no calls at merge (Fig. 2).

### Linkage disequilibrium, LD bins and tagSNPs

The pair-wise r^2^ values between merged SNP markers whose minor allele frequencies were at least 5% (common SNPs) and maximum inter-marker distance of 300 kb were calculated. LD bins were determined at threshold of r^2^ ≥ 0.80 by *TagZilla* version 1.0 (http://tagzilla.nci.nih.gov/). The program estimates LD bins using a greedy maximal approach similar to that of *ldSelect* [3]. As a result, 1,115,537 common SNPs were grouped in 366,214 LD bins, of which 189,417 were single-SNP bins. That left 17% of common SNPs without proxies. TagSNPs (representative SNPs for each bin) was selected by the *TagZilla* criteria “avesnp”, that is, having maximum average r^2^ with all other SNPs in the bin.

### CNV segments and CNV regions

B allele frequency (BAF) of heterozygous sites has been commonly used as an indicator of CNV of *Illumina* array data obtained from diploid materials. However, it is not an appropriate indicator in this study, because all SNPs in our duplicated haploid samples are expected to be genome-widely homozygous. And so, relative signal intensity of markers is the only variable for the detection of copy number changes, which we detected using circular binary segmentation algorithm implemented in the *R* statistical package module *DNAcopy 1.26* with default parameters [4]. Since the distributions of log2R and logRR were widely different, combined interpretation of the two data sets were inappropriate. Therefore, the segmentation analysis of the two data sets was carried out separately.

**Fig. 3:**
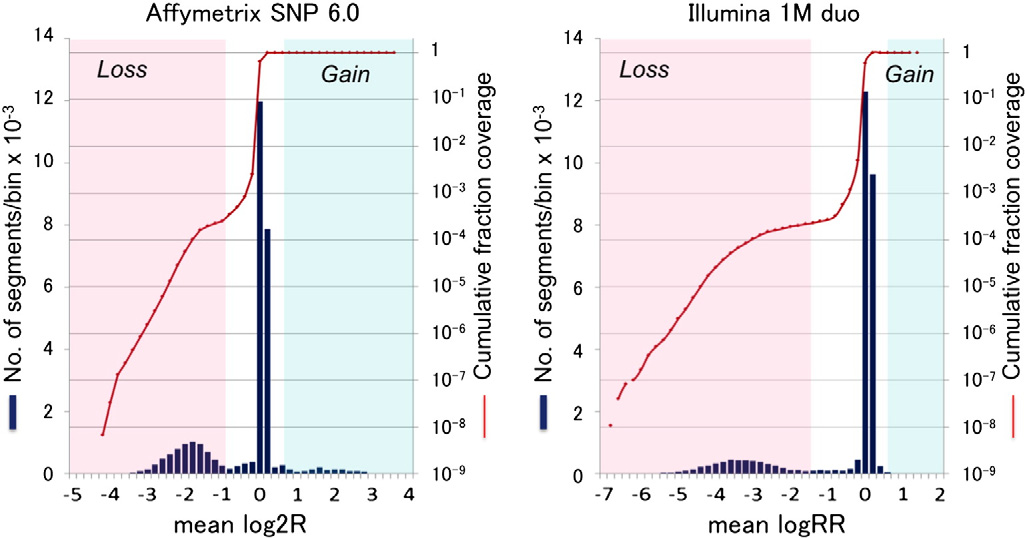
Distribution of copy number segments in bins of mean relative signal intensities. See text for detail.

Fig. 3 shows the distribution of mean relative signal intensities of segments defined by the two data sets (*Affymetrix* and *Illumina*). As shown in the figure, distinct peaks were observed in the regions below zero, apparently distinguishing deletion segments from normal copy segments. We defined the boundary of the two copy number states at the inflection points of cumulative segment coverage in each data set. Thus, the copy number states of segments having mean log2R < − 1 for *Affymetrix* and mean logRR < − 2 for *Illumina* were defined to be a loss, that accounted 0.02–0.03% of the genome. The thresholds for the definition of gain segments were not distinguishable from the plots, and we arbitrarily placed the boundary at 0.5 for both data sets. Then, a CNV segment that extended beyond centromere was split at the latter. The segments were filtered so that all of them had the sizes greater than 50 bp. The numbers of CNV segments defined by the two platforms are summarized in Table 1.

**Fig. 4:**
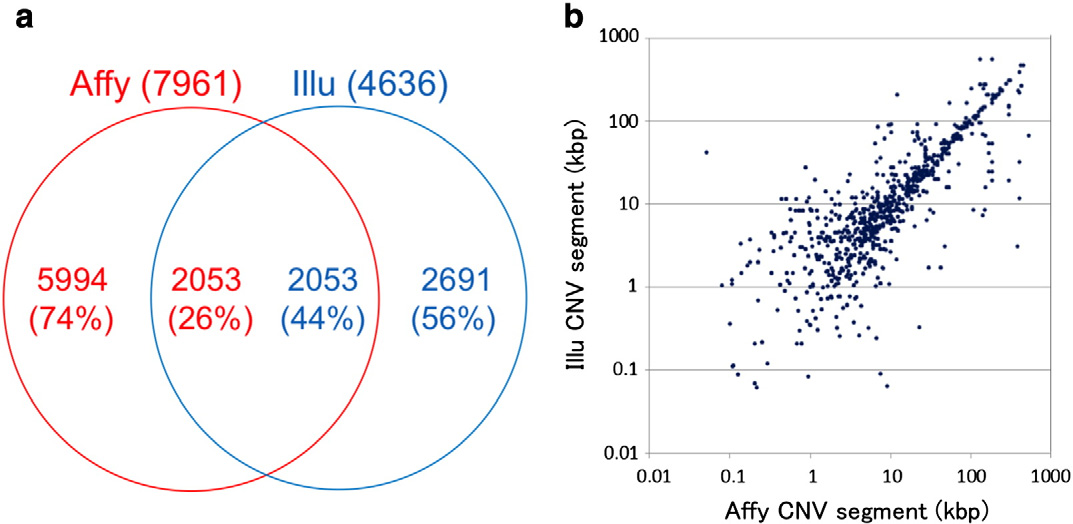
Overlap and size correlation of CNV segments detected by two platforms. a. The concordant calls of CNV segments between *Affymetrix* system and *Illumina* system were examined without distinguishing gains or losses, as detailed in the text. b. The lengths of overlapped CNV segments detected in the *Affymetrix* (abscissa) and *Illumina* (ordinate) systems are plotted.

The concordance of CNV segment calls between the two platforms was examined using an “intersect” function of *BEDTools* version 2.11.2 [5], setting a minimal overlap of one bp. The results revealed that in some genomic regions, mutually exclusive subsets of samples were judged to be in the CNV segments of opposite directions (gain by *Affymetrix* versus loss by *Illumina*). We also found that less than half of segments detected by the two arrays were overlapped (Fig. 4a). The reason for these apparent discrepancies should at least partly be attributable to the differences in the definition of reference intensities in the calculation of relative signal intensity and in the distribution of markers between the two systems, as discussed previously [1]. However, a good size correlation between overlapped segments was observed for segments longer than 10 kb, although some discrepancies by splitting/fusion of overlapped regions between the two platforms were observed even in long segments (Fig. 4b).

Next we defined CNV regions as merges of CNV segments across CHM samples without discriminating gains or losses. The results revealed a total of 2339 CNV regions that occupied 1.4% of the genome.

### Definitive Haplotype Database (D-HaploDB)

**Fig. 5:**
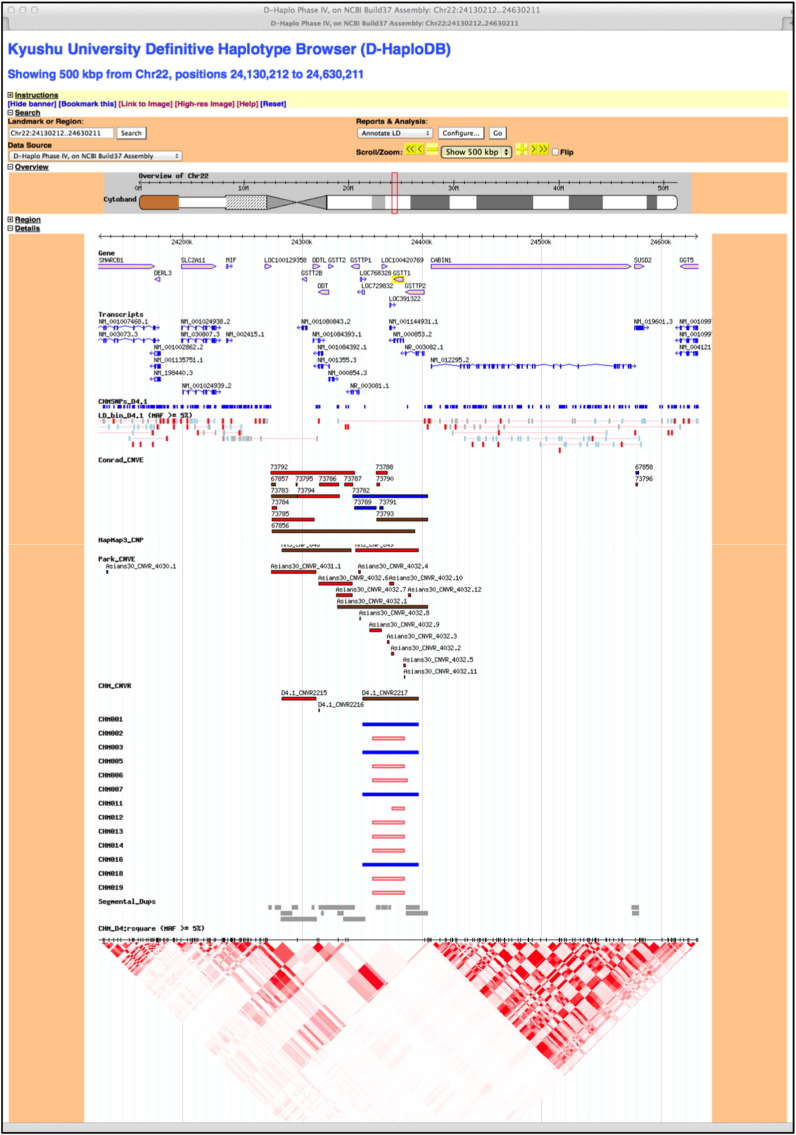
Screen capture of D-Haplo D4.1 glutathione S-transferase theta 1 region. CNV segments of gain or loss was detected by Affymetrix or Illumina systems, respectively, for mutually exclusive subsets of CHM samples. CNV segments of only a portion of samples are shown for the ease of viewing.

The results of SNP genotypings and CNV analyses described above are comprehensively presented in tracks (listed below) of *D-HaploDB* version 4.1 (http://orca.gen.kyushu-u.ac.jp) that uses *Generic Genome Browser* version 1.64 [6]. The genome coordinates are according to *GRCh37*. A screen shot of an example page of the database is shown in Fig. 5.

• CHMSNPs_D4.1: Merged SNPs genotyped using *Affymetrix* and *Illumina* platforms, and validated. Individual genotypes and allele counts are viewable by clicking the glyphs.
• Affymetrix SNP 6.0: Positions of *Affymetrix* markers are shown, with distinction of SNP probes (red) and CN probes (black).
• Illumina 1 M-duo: Positions of *Illumina* markers are shown, with distinction of SNP probes (red) and intensity only probes (black).
• LD_bin_D4.1 (MAF ≥  5%): The pair-wise r^2^ tagging at r^2^ ≥ 0.8 using *Tagzilla 1.0* program was done for SNPs whose minor allele frequencies were at least 5%. The best-tags (i.e., the tagSNP that showed the highest average r^2^ against the remaining members within the bin) are highlighted in red. Details containing SNP and haplotype information are viewable by clicking the glyphs.
• r-square (MAF ≥  5%): The r^2^ values from high to low between all combinations of markers within the selected regions are graphically shown by deep to shallow red.
• CHM_CNVR: CNV regions (CNVRs) in CHMs were defined as merges of CNV segments across all CHM samples. Thus, these are the regions where CNV segments were detected by either *Affymetrix* or *Illumina* platforms at least in one CHM.
• CHM#: CNV segments in each CHM sample (indicated by #) are shown with distinctions of losses (red) or gains (blue), and *Affymetrix* (dark) or *Illumina* (light).

In addition, some external data are incorporated and presented in tracks, to facilitate further interpretation of our data. Those are cytobands, genes, transcripts, segmental duplications and CNV data of Conrad et al. [7], HapMap3 [8], and Park et al. [9].

## Figures and Tables

**Table 1:** CNV segments defined by the two platforms.

Platform	Loss (per genome)	Gain (per genome)
Affymetrix SNP 6.0	6517 (78)	1444 (17)
Illumina 1 M-duo	4597 (55)	39 (0.5)

The definition of gain CNV segments is arbitrary. See text for detail.
